# IgG4-Related Sclerosing Cholangitis Involving the Intrahepatic Bile Ducts Diagnosed with Liver Biopsy

**DOI:** 10.1155/2018/2309293

**Published:** 2018-09-16

**Authors:** Malene Theilmann Thinesen, Ove B. Schaffalitzky de Muckadell, Sönke Detlefsen

**Affiliations:** ^1^Department of Pathology, Odense University Hospital, Odense, Denmark; ^2^Department of Gastroenterology, Odense University Hospital, Odense, Denmark

## Abstract

IgG4-related disease is characterized by lymphoplasmacytic inflammation and fibrosis, often leading to mass-forming lesions in different organs. When IgG4-related disease affects the bile ducts, it is called IgG4-related sclerosing cholangitis. A 74-year-old male complained of dysphagia and abdominal pain. Endoscopic retrograde cholangiography and magnetic resonance cholangiography revealed bile duct changes suspicious of a bile duct carcinoma or cholangitis. Liver biopsy showed storiform fibrosis, lymphoplasmacytic infiltration, obliterative phlebitis, and a portal-based inflammatory nodule with expansion of a portal tract. Hot spots revealed 339 IgG4-positive cells per high power field (HPF) and an IgG4/IgG ratio of 72%. Eight months earlier, an inguinal lymph node had been removed, showing expanded interfollicular zones and increased plasma cells. Hot spots revealed 593 IgG4-positive cells and an IgG4/IgG ratio of 92%. The serum IgG4 of the patient was elevated nearly 10 times upper limit of normal. The diagnosis of IgG4-related sclerosing cholangitis associated with IgG4-related lymphadenopathy was made. There was good response to treatment with prednisolone and azathioprine. The differentiation of IgG4-related sclerosing cholangitis from primary sclerosing cholangitis and bile duct carcinoma is often difficult. Liver biopsy only rarely contributes to this setting, but we describe and report in detail a case where liver biopsy showed a portal-based inflammatory nodule with the characteristic features of this disease.

## 1. Introduction

IgG4-related disease (IgG4-RD) has a broad spectrum of clinical manifestations, depending on which organ systems are involved. This disorder is characterized by fibro-inflammatory activity that may present as mass-forming lesions. The condition can affect one or multiple organs at the same time and is associated with elevated serum IgG4 in most cases [1–4]. The diagnosis of IgG4-RD depends on close collaboration between the treating physicians, the radiologist, and the pathologist. Histologically, lesions in IgG4-RD are characterized by a dense lymphoplasmacytic infiltrate, storiform fibrosis, and obliterative phlebitis. If several of these features are present and changes inconsistent with IgG4-RD are lacking, the diagnosis is further supported by documentation of increased numbers of IgG4-positive plasma cells as well as a ratio of IgG4- over IgG-positive cells above 40 % [1].

When IgG4-RD affects the bile ducts, it goes under the term IgG4-related sclerosing cholangitis (IgG4-SC). In some cases the differential diagnosis of IgG4-SC is complicated by its high degree of clinical and radiologic resemblance to primary sclerosing cholangitis (PSC) and bile duct carcinoma [5]. The current diagnostic criteria for IgG4-SC consist of cholangiography, serum IgG4, other organ manifestations of IgG4-RD, and characteristic histopathological features [6]. The task of differentiating between these entities is of high importance, as IgG4-SC responds to steroid treatment unlike PSC and bile duct carcinoma [2, 7]. In most cases of IgG4-SC, the characteristic histopathologic lesions are located in the hilar large bile ducts, with or without involvement of the common bile duct (CBD) or the intrahepatic bile ducts. Interestingly, in some IgG4-SC studies an expansive portal-based inflammatory nodule formed by fibrosis and inflammation has been recognized [7, 8]. This lesion is only identified in a minority of IgG4-SC cases at liver core needle biopsy (CNB), partly because involvement of the small peripheral bile ducts only occurs in about 20% of cases [2, 8]. With this case report, we aimed to present the liver biopsy features of IgG4-SC involving the small intrahepatic bile ducts, where liver biopsy was crucial in establishing the diagnosis.

## 2. Case Presentation

A 74-year-old male complained of dysphagia and abdominal pain lasting for two months. Esophagogastroduodenoscopy revealed esophageal candida infection that was treated with nystatin, a drug that has not been reported as causing hepatic injury. He had a history of arterial hypertension, prostatic hypertrophy, yet unspecified myopathy causing walking-disabilities for 40 years, and diabetes mellitus type 2 diagnosed four years prior to debut of the gastrointestinal symptoms. For the past 55 years he had smoked 20 cigarettes a day and had an alcohol consumption of 21 units (252 g) a week. Four and a half and one and a half years earlier, he had undergone surgical removal of a malignant melanoma Clark's level 2 from his left cheek and a basal cell carcinoma from his back. Two months earlier, a benign inguinal lymph node as well as nine colonic hyperplastic polyps had been removed. The lymph node was detected by physical examination as part of the follow-up program for malignant melanoma and was also seen at positron-emission tomography (PET).

**Figure 1:**
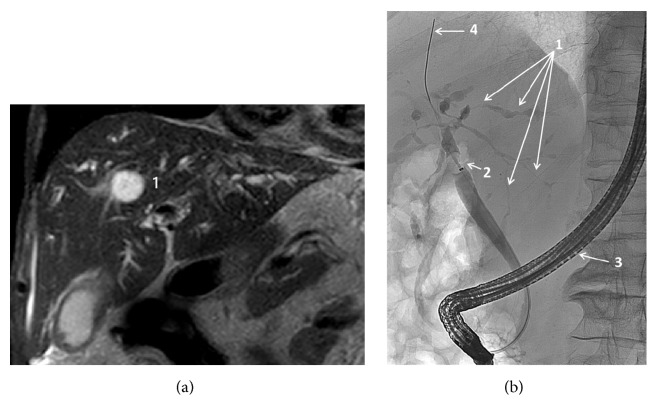
Imaging findings in a patient with IgG4-related sclerosing cholangitis (IgG4-SC). (a) At magnetic resonance imaging, no tumor-like lesions are seen. A benign hepatic cyst is indicated (1). (b) Diffuse and multiple intrahepatic biliary stenoses and dilatations at endoscopic retrograde cholangiography (1). The stenoses are relatively long. In classical primary sclerosing cholangitis (PSC), the stenoses would have been shorter, giving the cholangiogram a more beaded appearance. No complete obstruction of a bile duct is seen, a finding that would have been more characteristic of bile duct carcinoma. 2: occlusive balloon, 3: endoscope, 4: guide wire.

After treatment of the esophageal infection, his condition got worse and he developed jaundice as well as anemia. Dysphagia and abdominal pain continued and his appetite decreased. Furthermore, he developed weight loss, light-colored stools, dark-colored urine, diarrhea, and fatigue. An abdominal ultrasound showed gallbladder sludge, a poorly outlined and hypoechoic pancreas, and a dilated common bile duct, 8.7 mm in diameter. These findings aroused suspicion of gallstone-related cholecystitis. Additionally, abdominal contrast-enhanced computed tomography (c-CT) showed that the CBD had a diameter of 11 mm, intrahepatic cholestasis with stenosis at the hepatic duct bifurcation, a liver cyst located to segment 8, a right-sided renal tumor classified as Bosniak 3, pancreatic calcifications, and a presumed benign cyst located to the pancreatic neck. Serological tests revealed C-reactive protein (CRP) 79 mg/L [< 6 mg/L], hemoglobin 6.6 mmol/L [8,3-10,5 mmol/L], elevated alanine aminotransferase (ALT) 164 U/L [10-70 U/L], bilirubin 252 *μ*mol/L [5-25 *μ*mol/L], and alkaline phosphatase (AP) 500 U/L [35-105 U/L)]. The amylase was not elevated. The elevation of CRP was due to a urinary tract infection, and* E. coli* was isolated from the peripheral blood. He was treated with antibiotics, whereafter the CRP normalized and the hemoglobin almost normalized (7.4 mmol/L). After two failed attempts of endoscopic retrograde cholangiopancreaticography (ERCP), a magnetic resonance cholangiopancreaticography (MRCP) revealed a normal main pancreatic duct, intrahepatic cholestasis, and bile duct changes suspicious of a bile duct carcinoma Bismuth-Corlette type IV in the hilar region, involving both the right and left hepatic bile ducts. By magnetic resonance imaging (MRI), no hepatic tumors or pseudotumors but a benign cyst were observed (Figure 1(a)).

One month later, an ERCP showed multiple strictures of the small intrahepatic bile ducts in several liver segments, dilation of the CBD (12 mm), and a stenosis of the bifurcation (Figure 1(b)). Unfortunately, it was not possible to perform ERCP guided biopsy, for technical reasons. A stent was placed, and one week later, liver enzymes had improved: ALT 56 U/L, bilirubin 134 *μ*mol/L, and AP 343 U/L. Serum cancer associated antigen 19-9 (CA 19-9) was strongly elevated to 3003 kU/L [0-37 kU/L] but dropped to 165 kU/L after stent placement. Hereafter, either a bile duct carcinoma or PSC was suspected. Bile duct brush cytology revealed inflammation and atypical cells. Additional blood tests showed strongly elevated IgG4 (12.9 g/L, [0.052-1.40 g/L]), elevated IgG (25.69 g/L, [6.1-15.7 g/L]), negative cytoplasmic and perinuclear neutrophil cytoplasmic antibodies (c-ANCA and p-ANCA), and positive IgM rheumatoid factor. Serologic markers concerning viral hepatitis as well as anti-smooth-muscle antibodies, anti-liver-kidney microsome type 1 (LKM1) antibodies, anti-mitochondrial antibodies (AMA), glomerular basement membrane antibodies (GBM), liver cytosol specific antibody type 1 (anti-LC1), and anti-nuclear antibodies were all negative. He had a normal glomerular filtration rate (GFR).

**Figure 2:**
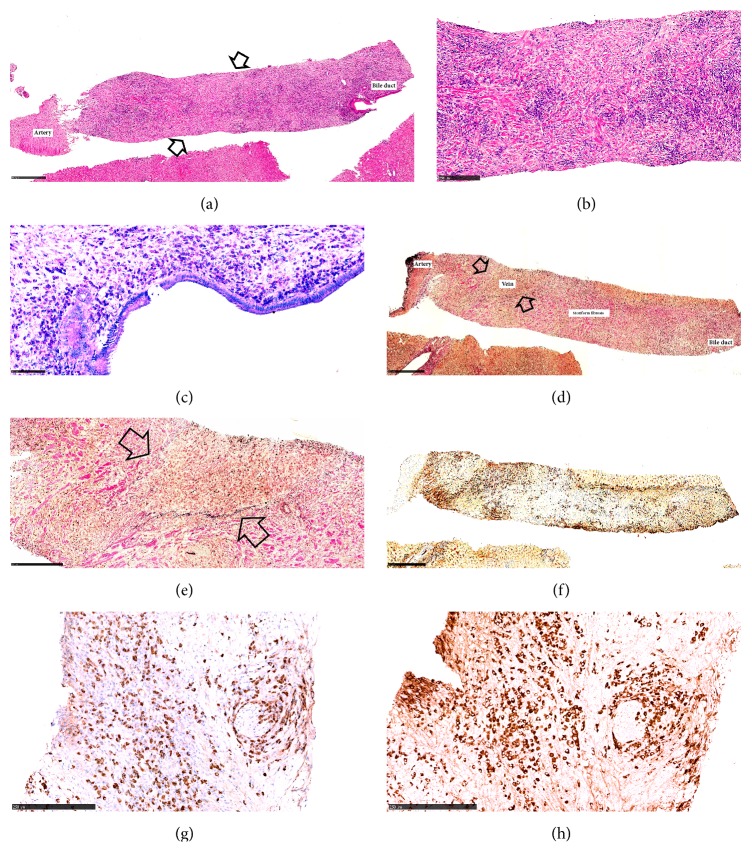
Liver core needle biopsy findings in a patient with IgG4-related sclerosing cholangitis (IgG4-SC). (a) A portal inflammatory nodule (IN), showing storiform fibrosis (arrows) and diffuse lymphoplasmacytic infiltration (H&E). (b) Higher magnification of the storiform fibrosis (H&E). (c) Strong infiltration of lymphocytes and plasma cells in the wall of the bile duct (Methyl-Green Pyronin). (d) Deeper section of the same area shown in Figure 2(a). At the periphery of the IN, there is obliterative phlebitis (arrows). Note the wall of the corresponding artery (Verhoeff elastin). (e) Higher magnification of the area with obliterative phlebitis shown in Figure 2(d). The former lumen of the vein is filled with plasma cells and lymphocytes. The vein can only be identified using an elastin stain (Verhoeff elastin). (f) Deeper sections of the area shown in Figure 2(a). Strong infiltration of IgG4-positive cells in the IN. (g, h) The IgG4 / IgG ratio is 72%.

To further investigate the possibility of an autoimmune etiology, particularly of IgG4-SC, a liver CNB was obtained (Figure 2). The liver biopsy had a length of 60 mm and contained 32 portal tracts. Twenty-four of the portal tracts showed chronic (mainly lymphoplasmacytic) inflammation, some of them with weak or moderate interphase activity. A portal-based, expansile inflammatory nodule (IN) measuring 6 mm in largest dimension, leading to expansion of a portal tract due to storiform fibrosis and lymphoplasmacytic infiltration, was identified (Figures 2(a) and 2(b)). Numerous myofibroblasts, immunohistochemically positive for smooth-muscle antigen, were observed in the IN (data not shown). No accumulation of neutrophilic granulocytes was found, and granulomas and multinucleated giant cells were lacking. In the bile duct located inside the IN, strong infiltration with lymphocytes and plasma cells was observed (Figure 2(c)). The bile duct mucosa was intact, without erosion or ulceration. The epithelium was mainly monolayered, but focally with slight hyperplasia. The epithelial cells were cylindric, and only focally slight variation of nuclear size was observed. Additionally, at some sites, a notable degree of obstructive cholestasis was found. Obliterative phlebitis was identified (Figures 2(d) and 2(e)) and, in addition, venolitis. Immunohistochemically, there was diffuse infiltration with IgG4-positive plasma cells (Figure 2(f)). Hot spots revealing 339 IgG4-positive and 468 IgG-positive plasma cells per high power field (HPF, 0.2 mm^2^) were found, corresponding to an IgG4/IgG ratio of 72% (Figures 2(g) and 2(h)). Moreover, microscopy showed several portal tracts with moderate periductal fibrosis and inflammatory infiltrates dominated by lymphocytes and plasma cells and associated with eosinophilic granulocytes (in hot spots up to 13 /HPF). In the light of these findings, the diagnosis of IgG4-SC involving the extra- and intrahepatic small bile ducts was suggested. At this time, it was speculated whether the patient also had IgG4-positive autoimmune pancreatitis (AIP) type 1, another manifestation of IgG4-RD that is often associated with IgG4-SC. However, the main pancreatic duct was unremarkable by MRCP, the amylase was not elevated, no focal or diffuse enlargement of the pancreas or delayed enhancement was found at c-CT, and calcifications and cysts are usually not a feature of AIP. Hence, the International Consensus Diagnostic Criteria (ICDC) for AIP were not fulfilled [9]. Other lesions frequently associated with IgG4-SC are IgG4-related retroperitoneal fibrosis and IgG4-related thyroiditis, but these manifestations were not present in our patient [10, 11].

**Figure 3:**
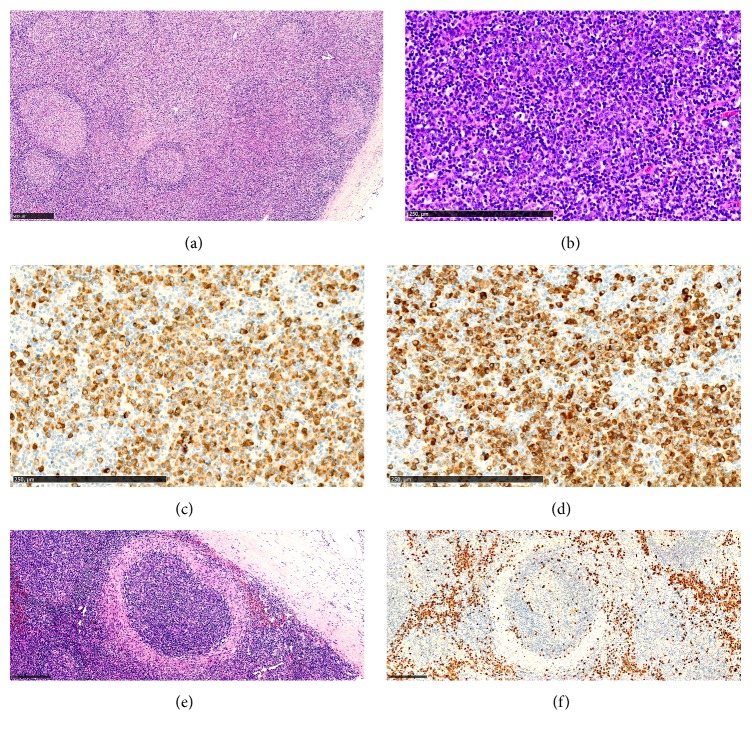
Histological findings in a patient with IgG4-related lymphadenopathy. (a) Low magnification showing expansion of the interfollicular zone (H&E). (b) At higher magnification, numerous plasma cells are identified in the interfollicular zones (H&E). (c, d) Strong infiltration of IgG4-positive cells, hot spot (c) (IgG4 immunostaining) and IgG-positive cells, hot spot (d) (IgG immunostaining). The IgG4 / IgG ratio is 92%. (e) A granulomatous focus, arranged in a ring-like fashion around a lymphoid follicle, is shown, a feature called perifollicular granuloma (H&E). (f) Another perifollicular granuloma, accompanied by numerous IgG4-positive cells (IgG4 immunostaining).

The inguinal lymph node that was surgically removed two months before debut of the gastrointestinal symptoms was initially classified as reactive with nodular lymphoid hyperplasia and smaller areas with nonnecrotic granulomatous inflammation. The lymph node was retrieved from the archive and showed expanded interfollicular zones as well as follicular hyperplasia with activated germinal centers (Figure 3(a)). Interfollicular zones as well as follicles were infiltrated by an increased number of plasma cells (Figure 3(b)). In several foci, the germinal centers were penetrated by small venules. Immunohistochemically, hot spots revealing 593 IgG4-positive and 646 IgG-positive plasma cells per high power field (HPF, 0.197-0.199 mm^2^) were found, corresponding to an IgG4/IgG ratio of 92% (Figures 3(c) and 3(d)). Based on the above, it was assumed that the patient had IgG4-related lymphadenopathy associated with IgG4-SC. Interestingly, there were a few smaller areas with a characteristic granulomatous inflammation. These granulomatous foci were arranged in a ring-like fashion around lymphoid follicles, a feature also called perifollicular granuloma (Figure 3(e)). Epitheloid granulomas usually make a diagnosis of IgG4-RD unlikely [1]. However, perifollicular granulomas are an exception, as they have been reported in a number of cases of IgG4-related lymphadenopathy [12–15].  Figure 3(f) shows that the perifollicular granulomas were accompanied by numerous IgG4-positive cells. Of note, perifollicular granulomas are not specific for IgG4-related lymphadenopathy and can also be observed in, for example, nodular lymphocyte predominance Hodgkin lymphoma or reactive lymph nodes of unknown etiology [12].

The patient began combined treatment with a daily dose of 100 mg azathioprine and 37.5 mg prednisolone. Within one month, the daily dose of prednisolone was tapered to 12.5 mg. Liver enzymes and AP decreased further after initiation of medical treatment. The dose of prednisolone was whatsoever not tapered continuously due to his muscle disease that seemed to improve due to steroids. The patient stopped with prednisolone 16 months after initiation of the immunosuppressive treatment and now, 26 months later, he is stable without recurrence, taking 150 mg azathioprine daily. However, at present, it is considered to increase his insulin dose, as the blood glucose levels are suboptimal. Unfortunately, no follow-up imaging was performed. Instead, AP, ALT, and immunoglobulins were checked regularly and are currently normal.

## 3. Discussion

The case presented here emphasizes that clinically and cholangiographically, IgG4-SC resembles bile duct carcinoma and PSC in many aspects. The diagnostic process is therefore based on close cooperation between the treating gastroenterologist, surgeon, radiologist, and pathologist. Due to its usually good response to steroid treatment and the dismal prognosis of its two main differential diagnoses, the correct diagnosis of IgG4-SC is of high importance. Frequently, IgG4-SC is associated with autoimmune pancreatitis (AIP) type 1 or other manifestations of IgG4-RD [16, 17]. Clinically and demographically, patients with IgG4-SC are mainly middle-aged to elderly males, debuting with obstructive jaundice, weight loss, steatorrhea, and abdominal pain, similarly to extrahepatic/hilar bile duct cancer [2, 18]. Serum IgG4 is elevated in most cases of IgG4-SC but is not a fully reliable adjunct in the differential diagnosis, as it also may occur in some patients with PSC and cancer and as its sensitivity is only around 90% [19, 20]. Cholangiographically, IgG4-SC also imitates PSC or bile duct carcinoma, as in the case presented here [21]. However, the diffuse distribution of the bile duct changes in our case was more suggestive of an inflammatory disease than bile duct carcinoma, and there was no complete obstruction. A focal concentric bile duct wall thickening with dilatation of the peripheral bile ducts, as well as complete obstruction of a bile duct, had been more suggestive of bile duct carcinoma [22, 23]. Cholangiographically, IgG4-SC was more likely than PSC because the stenoses were relatively long, while in classical PSC, the strictures are usually more band-like and shorter than the dilated segments, leading to a beaded appearance [24]. An IgG4-SC study by Naitoh et al. indicated that involvement of the peripheral small intrahepatic bile ducts correlates with intrahepatic biliary strictures at ERCP [8]. In these cases, there is a higher probability that a liver CNB will yield features diagnostic of IgG4-SC [8]. A liver CNB likely diagnostic of IgG4-SC must show at least two of the three “Boston criteria”: storiform fibrosis, obliterative phlebitis and dense lymphoplasmacytic infiltration, and the absence of features advocating against this diagnosis, such as malignancy, necrosis, or multinucleated giant cells [1]. Increased IgG4-positive plasma cells (in a liver CNB at least 10 IgG4-positive plasma cells/HPF) and an increased IgG4/IgG ratio (>40%) can further support a diagnosis of IgG4-SC [1, 6]. The diagnostic criteria for IgG4-SC, as published by the Japanese Working Group, include characteristic biliary imaging findings, elevated serum IgG4, other organ manifestations of IgG4-RD, and characteristic histopathological features [6]. The current case report also met these diagnostic criteria. A study by Chen and Deshpande, aimed at differentiating between IgG4-SC and PSC based on liver CNB, reported that an expansile portal-based inflammatory nodule (IN) was found in half of the IgG4-SC cases (n=10) but in none of the PSC cases (n=17) [7]. The IN was composed of lymphocytes, plasma cells, eosinophils, fibroblasts, and a storiform fibrosis, similar to the case presented here [7]. Naitoh reported an IN in a liver CNB from a patient with IgG4-SC (one of 19 IgG4-SC patients) [8]. In the present case, we identified a portal vein and a hepatic artery in the IN. Hence, it is unlikely that the IN represented an IgG4-positive inflammatory pseudotumor (IgG4-IPT), another IgG4-related lesion that can occur in the liver [25]. In patients without cholangiographic intrahepatic bile duct strictures but involvement of the extrahepatic bile ducts, a cholangioscopic bile duct forceps biopsy can sometimes be of diagnostic value [26]. However, in patients who underwent stenting prior to bile duct biopsy, reactive epithelial changes may be present that can imitate neoplastic changes. The hallmarks of IgG4-SC, namely, storiform fibrosis, obliterative phlebitis, and increased IgG4-positive cells, are often located deep in the bile duct wall and may therefore often be missed in bile duct biopsy specimens, which are often superficial and tiny and only contain limited amounts of subepithelial connective tissue. The mere presence of subepithelial lymphoplasmacytic infiltrates showing IgG4-positivity may support a diagnosis of IgG4-SC in some instances, but can also be present in cases of bile duct cancer and even PSC [27, 28]. Hence, for the biopsy diagnosis of IgG4-SC, as for other organ manifestations of IgG4-RD, a frequent limitation is the scarcity of the tissue specimens available for the diagnosis, which may prevent a clear-cut diagnosis [5].

IgG4-related lymphadenopathy is both an “under- and overdiagnosed entity”, as mentioned by Cheuk et al. [12]. Five different histological patterns (I-V) have been reported as part of its morphological spectrum [12]. Most of the features overlap and are present in more than just one of the reported patterns, although the interfollicular expansion found in our case seems to be present in only pattern III [12]. Due to the low specificity of the morphological features in IgG4-related lymphadenopathy, as obliterative phlebitis and storiform fibrosis are often not present, an IgG4/IgG ratio > 40% and more than 100 IgG4-positive cells per HPF are essential for its diagnosis [12]. However, many other conditions, such as Rosai-Dorfman disease, multicentric Castleman disease, or rheumatoid arthritis, may show increased IgG4-positive cells and an increased IgG4/IgG ratio [12]. Therefore, an increased serum IgG4 value and/or extranodal manifestations of IgG4-RD should be present [12]. The current case shows both the morphological characteristics and meets the threshold of IgG4/IgG ratio and IgG4-positive cell count. Besides, the patient developed symptoms of IgG4-SC two months after surgical removal of the lymph node, and at that time, he had a serum IgG4 value ten times above upper limit of normal. Therefore, the diagnosis of IgG4-related lymphadenopathy seemed reasonable in our patient.

Our patient was diagnosed with malignant melanoma and basal cell carcinoma 4.5 and 1.5 years prior to the development of IgG4-RD. It is at present not clear whether IgG4-RD may be a form of paraneoplastic syndrome in patients with malignancies. A few follow-up studies indicate an increased risk for the development of malignancies in IgG4-RD, including patients with type 1 AIP and IgG4-SC [29–31]. However, a Japanese study from 2014 reported no increased incidence in total number of malignancies between patients with IgG4-RD and the general population [32]. Two studies based on European patients reported a relatively high cumulative frequency of malignant diagnoses prior to or after a diagnosis of type 1 AIP (14.3% and 25%) [33, 34]. Further studies, however, are needed to elucidate the risk of malignancies in IgG4-RD.

The patient presented here had unspecified myopathy and noticed a marked improvement related to treatment of IgG4-RD with steroids. It is well known that inflammatory myopathy responsive to steroids can develop in association with autoimmune diseases such as systemic sclerosis, systemic lupus erythematosus, Sjögren's syndrome, and rheumatoid arthritis, a condition termed overlap myositis [35]. Hence, one may speculate whether there may be a pathophysiologic relation between IgG4-RD and the myopathy in our patient. Unfortunately, no muscle biopsy, the gold standard for diagnosing overlap myositis, was obtained in our patient [35]. However, to our knowledge, autoimmune liver and bile duct diseases are only very rarely associated with myopathies. Besides, apart from IgG4-related orbital myositis, myositis is not a well described manifestation of IgG4-RD [1]. Recently, in a patient diagnosed with polymyositis, elevated serum IgG4 and infiltration of IgG4-positive cells in the left quadriceps muscle were reported, and it was concluded that polymyositis may be a mimicker of IgG4-RD [36]. Hence, it is most likely that the myopathy in our patient was unrelated to IgG4-RD.

In conclusion, we present a case of a male with IgG4-related sclerosing cholangitis involving the small intrahepatic as well as the hilar bile ducts, initially suspected to represent bile duct cancer, diagnosed with liver biopsy, and associated with IgG4-related lymphadenopathy.
